# Identification of biomarkers associated with proliferation and differentiation of mesenchymal stem cells in pulmonary adenocarcinoma and establishment of prognostic models

**DOI:** 10.1186/s41065-025-00492-7

**Published:** 2025-07-01

**Authors:** Xin-Xin Zeng, Ke-Xin Xian, Jie-Lun Wen, Qi-Zhe Wang, Xin-Yu Wang, Li-Yue Sun

**Affiliations:** 1 Second Department of Oncology, Guangdong Second Provincial General Hospital, Guangzhou, China; 2 Department of Radiation Oncology, Guangdong Medical University, Zhanjiang, China; 3 School of Medicine, Jinan University, Guangzhou, China; 4 Department of Health Management Centre, Zhongshan Hospital, Fudan University, Shanghai, China; 5 Department of General Practice, Zhongshan Hospital, Fudan University, Shanghai, China

**Keywords:** Lung adenocarcinoma, Mesenchymal stem cells, Prognosis, TCGA, Immune infiltration

## Abstract

**Background:**

Mesenchymal stem cells (MSCs) hold potential as therapeutic agents in cancer, but their mechanisms in lung adenocarcinoma (LUAD) remain poorly understood. This study aimed to identify biomarkers associated with MSC proliferation and differentiation (MSCPD) and investigate their regulatory roles in LUAD.

**Methods:**

Using the TCGA-LUAD and GSE72094 datasets, MSCPD-related gene (MSCPD-RG) scores were calculated, and samples were divided into high and low subgroups. Differentially expressed genes (DEGs1: between subgroups; DEGs2: tumor vs. normal) and module genes derived from weighted gene co-expression network analysis (WGCNA) were examined. Overlapping genes were subjected to Cox and LASSO regression to identify potential biomarkers. A prognostic risk model was developed and validated, followed by functional, immune, and drug sensitivity analyses.

**Results:**

Four biomarkers (MS4A2, IGSF10, NTRK3, MFAP3L) were identified from 1,061 DEGs1, 6,604 DEGs2, and 610 module genes. The risk model based on these biomarkers accurately stratified prognosis. Both T stage and risk score were independent prognostic factors, and a nomogram integrating these factors demonstrated high predictive accuracy. These biomarkers were notably enriched in pathways related to ribosome function, cell cycle regulation, and oxidative phosphorylation. Immune cell analysis revealed significant differences in nine immune cell types (e.g., plasma cells, CD4 memory T cells) between LUAD and normal tissues.

**Conclusion:**

In this study, four key biomarkers closely related to mesenchymal stem cell proliferation/differentiation (MSCPD) were identified in lung adenocarcinoma (LUAD), namely MS4A2, IGSF10, NTRK3, and MFAP3L. Through multi-omics integrated analysis and independent cohort validation, it was confirmed that these markers not only affect disease progression by regulating mesenchymal - epithelial transition (MET) and tumor microenvironment remodeling but can also effectively predict patient prognosis and response to immunotherapy.

**Supplementary Information:**

The online version contains supplementary material available at 10.1186/s41065-025-00492-7.

## Introduction

Lung adenocarcinoma (LUAD), a major subtype of non-small cell lung cancer (NSCLC), i has both high incidence and mortality [1]. Globally, LUAD results in an average annual death toll exceeding 1.8 million. According to the latest 2024 statistics from the American Cancer Society, deaths from all types of lung cancer in the United States are estimated at approximately 125,070 [2]. LUAD typically presents as peripheral nodules or masses, often asymptomatic in its early stages, and is frequently diagnosed at advanced stages [3]. Smoking, air pollution, and radiation are widely regarded as primary environmental risk factors for LUAD [4]. In addition, LUAD is linked to the aberrant activation of key driver genes such as EGFR [5], ALK [6], and KRAS [7]. LUAD has undergone several revolutionary shifts. The introduction of chemotherapy in the 1890s established the precedent for systemic anticancer therapy. The breakthrough of targeted therapy in the early 21st century advanced cancer treatment from “indiscriminate killing” to precision medicine. More recently, progress in immunotherapy has further refined the treatment paradigm towards precise modulation of the tumor microenvironment [8]. Consequently, the current therapeutic focus has shifted from solely targeting cancer cells to co-regulating the tumor microenvironment– studies indicate that adjuvant therapies targeting tumor-associated fibroblasts or immune cells significantly improve patient treatment response rates [9]. This evolution aligns closely with the epidemiological characteristics of LUAD. Despite continuous advancements in targeted therapy, immunotherapy, and novel treatments [10], the 5-year survival rate for LUAD remains low [11], and patients frequently develop drug resistance within months of starting medication [12]. LUAD treatment is thus experiencing a paradigm shift from traditional models towards precision medicine. Future strategies will focus on three key directions: (1) personalized combination therapies based on multi-omics, (2) adaptive treatment guided by spatiotemporal dynamic monitoring, and (3) artificial intelligence-driven treatment optimization [13]. Regarding mesenchymal stromal cells (MSCs) regulation in LUAD, the emerging “cell niche reconstruction” strategy offers potential to overcome current drug resistance by simultaneously targeting tumor stem cells and their microenvironmental niches. Consequently, identifying novel biomarkers that can predict prognosis and guide treatment decisions holds significant promise in improving patient outcomes and reducing the burden of this devastating disease.

Mesenchymal stem cells are multipotent stromal cells capable of differentiating into various cell types, including osteoblasts, chondrocytes, myocytes, and adipocytes [14]. They play a pivotal role in maintaining tissue homeostasis and facilitating repair, primarily through their proliferative and differentiative capacities. Additionally, MSCs are implicated in the regulation of cancer progression, including LUAD, through mechanisms such as immune modulation, angiogenesis, and direct interaction with tumor cells [15]. Moreover, the relationship between MSC proliferation and differentiation and the development of LUAD has been the subject of several studies [16]. Research indicates that MSCs can accelerate LUAD progression by enhancing tumor cell proliferation, invasion, and metastasis [17]. Furthermore, MSCs contribute to the formation of the tumor microenvironment, which plays a critical role in LUAD progression [16]. Although some biomarkers associated with MSC proliferation and differentiation have been identified, the precise molecular mechanisms by which these markers regulate MSC behavior in LUAD remain elusive. Furthermore, the details of the interactions between MSCs and immune cells within the tumor microenvironment remain incompletely characterized.

In recent years, the role of MSCs in the tumor microenvironment has garnered increasing attention, yet the precise regulatory mechanisms and prognostic value of their proliferation and differentiation in LUAD remain incompletely characterized. Current research predominantly focuses on individual MSCPD-related genes’ functions within the tumor microenvironment– such as immune regulation and angiogenesis– or analyzes single-gene prognostic value using isolated datasets [18]. This approach reveals a critical gap: systematic screening of MSCPD-related genes and exploration of their multidimensional mechanisms across integrated datasets remain lacking.In this study, transcriptome data from 497 patients with LUAD, including survival and clinical information, and 58 normal controls from The Cancer Genome Atlas (TCGA) database were analyzed. Using bioinformatics techniques such as differential gene expression analysis and machine learning, this study identified four MSC proliferation- and differentiation-related biomarkers—MS4A2, IGSF10, NTRK3, and MFAP3L—in LUAD for the first time. Additionally, the roles of these biomarkers in immune response regulation and signaling pathways involved in LUAD were investigated. These findings offer insights into the potential functions and molecular mechanisms of these biomarkers in LUAD. This study not only enhances our understanding of LUAD pathogenesis but also provides potential targets for the diagnosis and treatment of this malignancy.

## Materials and methods

### Data source

The LUAD dataset was obtained from the TCGA database (https://portal.gdc.cancer.gov/), which included 497 LUAD samples with survival and clinical data, as well as 58 control cases. The GSE72094 dataset (GPL15048) and GSE31210 dataset (GPL570 were retrieved from the Gene Expression Omnibus (GEO) database (http://www.ncbi.nlm.nih.gov/geo/), respectively include 442 and 226 LUAD samples. The Molecular Signatures Database (MSigDB, http://www.gsea-msigdb.org/gsea/index.jsp) provided a list of 44 MSC Proliferation- and Differentiation-related genes (MSCPD-RGs). These included the gene sets GOBP MESENCHYMAL STEM CELL DIFFERENTIATION, GOBP MESENCHYMAL STEM CELL MAINTENANCE_INVOLVED IN NEPHRON MORPHOGENESIS, GOBP MESENCHYMAL STEM CELL PROLIFERATION, GOBP POSITIVE REGULATION OF MESENCHYMAL STEM CELL PROLIFERATION, and GOBP REGULATION OF MESENCHYMAL STEM CELL DIFFERENTIATION, which contained 12, 6, 11, 7, and 8 MSCPD-RGs, respectively, yielding a total of 27 MSCPD-RGs after de-duplication.

### Data processing and batch effect correction

This study adopted a rigorous data processing pipeline to perform standardized analysis on the transcriptomic data of lung adenocarcinoma from the TCGA and GEO databases. First, TCGA data were normalized using the DESeq2 package (v1.42.0) [19]via the DESeq() function, while GEO data underwent quantile normalization using the limma package (v3.58.1) [20]. To eliminate batch effects, the ComBat algorithm from the sva package (v3.50.0) was applied for data correction.

### Weighted gene co-expression network analysis (WGCNA)

To identify LUAD-associated genes, TCGA-LUAD samples were analyzed using WGCNA (v1.71) [21]. After excluding outliers, the optimal soft threshold (β) was selected to achieve scale-free topology (R^2^ close to 0.9 ). Modules were identified via dynamic tree cutting (min genes = 150), with key modules selected based on strong disease correlation (*P* < 0.001). Significant module gene were filtered by|MM|>0.6 and|GS|>0.2 [22].

### Selection and function enrichment of candidate genes

For TCGA-LUAD, MSCPD-RGs scores for all cases were calculated using the single-sample gene set enrichment analysis (ssGSEA) algorithm in GSVA (v 1.44.5) [23]. Inter-group differences were assessed using the Wilcoxon rank-sum test (*P* < 0.05). The LUAD samples were then divided into high and low scoring groups based on the MSCPD-RGs scores using the surv cutpoint function in the survminer package (v 0.4.9) [24] (minprop = 0.4). Minprop = 0.4 indicates that when the samples are divided into high-risk and low-risk groups, the number of samples in each group accounts for at least 40% of the total sample siz. Kaplan-Meier (KM) survival curves, derived from the Survival package (v 3.4-0) [25], were employed to compare overall survival (OS) between the two groups (Fleming-Harrington test, *P* < 0.05).

In TCGA-LUAD, differential expression analysis was performed using the DESeq2 package (v 1.36.0) to identify differentially expressed genes (DEGs1) between high and low scoring groups, and DEGs2 between LUAD and control groups, with significance defined as|log_2_ fold change (FC)| > 0.5 and *P.adj* < 0.05 [19]. Expression of DEGs was visualized using volcano plots and heatmaps generated with the ggplot2 (v 3.3.6) and pheatmap (v 1.0.12) packages [26, 27]. Finally, DEGs1 and DEGs2 were intersected with key module genes using the ggvenn package (v 0.1.9) (https://CRAN.R-project.org/package=ggvenn) to identify candidate genes in TCGA-LUAD.

### Candidate genes function exploration and protein-protein interaction network (PPI) construction

To explore the biological functions potentially associated with the candidate genes, Gene Ontology (GO) and Kyoto Encyclopedia of Genes and Genomes (KEGG) analyses were performed using the clusterProfiler package (v 4.7.1.001) (*P* < 0.05) [28]. The top eight GO and KEGG terms were displayed in descending order of *P*-value, from smallest to greatest, using the ggpubr (v 0.5.0) and GOplot (v 1.0.2) programs [26, 29]. Additionally, the Search Tool for the Retrieval of Interacting Genes/Proteins (STRING, https://string-db.org/) database was used to analyze the PPI network of the candidate genes [30]. For visualization, interaction data were imported into Cytoscape (v 3.8.2) [31].

### Cox regression and least absolute shrinkage and selection operator (LASSO)

To identify genes significantly associated with survival, univariate Cox regression analysis was conducted on LUAD cases in TCGA-LUAD, selecting genes with a Hazard Ratio (HR) ≠ 1 and *P* < 0.05 using the survival package (v 3.4-0). Proportional hazards (PH) assumption testing was then performed on eligible genes with *P* > 0.05. Genes that passed both the PH assumption test and the univariate Cox test were subsequently analyzed using LASSO regression with the glmnet package (v 4.1-6) and multivariate Cox regression analysis with HR ≠ 1 and *P* < 0.2 as inclusion criteria to identify biomarkers [32].

### Construction and validation of risk model

The risk score was calculated, and a risk model was constructed using the following formula, which incorporated the biomarkers’ expression and associated survival data.

$$risk\:score = \sum\nolimits_{i = 1}^n {\left( {coe{f_i}*{X_i}} \right)} $$

In this formula, Coef and X indicated coefficients and gene expression, respectively. The 497 patients with LUAD were divided into high-risk and low-risk groups based on the median value of the risk score. The pheatmap (v 1.0.12) was used to visualize biomarker expression differences between the two groups. The KM survival curve was generated using the survival package (v 3.5-3) to compare OS between the groups (log-rank test, *P* < 0.05). Additionally, the 1, 3, and 5-year receiver operating characteristic (ROC) curves were plotted using the survivalROC package (v 1.0.3) to assess the model’s predictive performance (area under the curve (AUC) > 0.6) [33]. The risk model was validated in the GSE72094 dataset and GSE31210 dataset in the same manner (with the survival analysis threshold set at *P* < 0.05) to assess its generalizability.

### Clinical profiling and nomogram construction

To evaluate independent prognostic factors, clinical characteristics (age, gender, stage, T, N, M) and risk scores were included in both univariate and multivariate Cox regression analyses, as well as PH assumption testing (*P* > 0.05). A nomogram was developed based on independent prognostic factors using the rms package (v 6.3-0) to predict 1-, 3-, and 5-year survival rates [34]. To assess the reliability of this nomogram, calibration curves for the 1-, 3-, and 5-year time points were created using the rms package (v 6.3-0). Additionally, ROC curves for 1-, 3-, and 5-year time spans were plotted using the survivalROC package (v 1.0.3) to evaluate the nomogram (AUC > 0.7). Finally, to assess the clinical effectiveness of the nomogram, decision curve analysis (DCA) curves for 1-, 3-, and 5-year survival were constructed using the rmda package (v 1.6) (https://github.com/mdbrown/rmda).

### Functional exploration in LUAD

To further investigate the underlying biological mechanisms between the two risk groups, GSEA was performed using the clusterProfiler package (v 4.7.1.001) [28]. Initially, differential expression analysis was conducted between the two risk groups using DESeq2 (v 1.36.0), with|log_2_ FC| values used to rank the differential genes. The background set for enrichment analysis was chosen as c2.cp.kegg from the MSigDB database, with enrichment criteria set as|normalized enrichment score (NES)| > 1 and *P.adj* < 0.05.

### Immune infiltration analysis

To assess immune cell infiltration differences between the two risk groups, the proportions of 22 immune cell types were calculated for LUAD samples using the CIBERSORT algorithm (v 1.03) [35], with the LM22 gene set [35]. Inter-group differences were then compared, excluding samples with *P*-values greater than 0.05 or infiltration proportions of 0 cells. Bar plots representing immune cell infiltration abundance were generated using the ggplot2 (v 3.3.6) package [26]. Spearman correlations (*P* < 0.001) between biomarkers and differential immune cells were calculated using the psych package (v 2.2.9) (https://CRAN.R-project.org/package=psych).

### Competing endogenous RNA (ceRNAs) network construction

To explore the molecular regulatory mechanisms of the biomarkers in LUAD, ceRNA networks were constructed. First, differentially expressed miRNAs (DE-miRNAs) and long non-coding RNAs (DE-lncRNAs) in TCGA-LUAD were identified using the same R packages and thresholds applied in mRNA differential analysis, and volcano plots were created for visual representation. Predicted miRNAs regulating the biomarkers were retrieved from the starBase database (starbase.sysu.edu.cn) (with at least 2 mRNA-miRNA interactions). These predicted miRNAs were then intersected with the DE-miRNAs using the ggvenn package (v 0.1.9). The miRNAs that exhibited the opposite expression trend of the biomarkers in the intersection were identified as target miRNAs. Following this, target lncRNAs were similarly identified, and the ceRNA network was constructed and visualized using Cytoscape (v 3.8.2) [31].

### Chemotherapy drug sensitivity analysis

To assess the therapeutic effects of chemotherapeutic agents in patients with LUAD, a total of 198 chemotherapeutic agents were retrieved from the Genomics of Drug Sensitivity in Cancer (GDSC) database (https://www.cancerrxgene.org/). The half-maximal inhibitory concentration (IC_50_) for each drug was evaluated using the oncoPredict package (v 0.2) [36], based on LUAD samples from the training set. Differences in IC_50_ values between the two risk groups were assessed using the Wilcoxon test (*P* < 0.05).

### Tissue specimen collection

LUAD tissue and normal lung tissue samples were obtained from lung cancer resection surgeries at Guangdong Second Provincial General Hospital in 2023. The samples were fixed in 10% neutral formalin and embedded in paraffin for further analysis. The study received approval from the Ethics Committee of Guangdong Second Provincial General Hospital (2022-KZ-295), and written informed consent was obtained from all patients.

### DNA extraction and quantitative real-time PCR

DNA was extracted from 9 LUAD tissues and 36 normal lung tissues using the FFPE DNA extraction kit (TianGen Biochemistry, Beijing). Quantitative real-time PCR (qRT-PCR) was performed using the TaqMan probe method (ABI QuantStudio 6, Applied Biosystems, CA). Reactions were conducted in 96-well plates in triplicate, with each well containing FAM-labeled and VIC-labeled probes for four target genes and a Cy5-labeled probe for the reference gene GAPDH. Furthermore, the reaction mixture included 10 ng of tissue DNA, 7.5µL of the mixture and 0.64µL of primers. Cycling conditions consisted of one cycle at 95 °C for 5 min, followed by 20 cycles at 95 °C for 15 s, 64 °C for 30 s, and 40 cycles at 60 °C for 10 s. Relative gene expression levels were calculated using the 2^−△△ct^ method.

### Statistical analysis

The bioinformatics analysis was performed in R language (v4.2.2). DESeq2 (v. 1.36.0) [19] was used for differential expression analysis to explore the differences in gene expression between lung adenocarcinoma and control samples; WGCNA (v 1.71) [21] was used to conduct weighted gene co - expression network analysis to explore the association between gene modules and disease phenotypes; GSVA (v 1.44.5) [23] was used for score calculation to quantify the activity of gene sets; survminer (v 0.4.9) (https://CRAN.R-project.org/package=survminer).was used for survival analysis to analyze the prognostic influencing factors; clusterProfiler (v 4.7.1.001) [28] was used to jointly carry out enrichment analysis to explain the enrichment rules of gene functions; Cytoscape (v 3.8.2) [35] was responsible for network drawing to present the gene - protein interaction and other network relationships; glmnet (v 4.1–6) [32] was used for Lasso analysis for feature selection; rms (v 6.3–0) (https://CRAN.R-project.org/package=rms) was used to construct nomograms to realize the visualization of clinical prediction models; psych (v 2.2.9) (https://CRAN.R-project.org/package=psych) was used to calculate correlations; CIBERSORT (v 1.03) [35] was used to analyze the immune infiltration situation to reveal the tumor immune microenvironment; oncoPredict (v 0.2) (https://CRAN.R-project.org/package=oncoPredict>) was used to evaluate the sensitivity to chemotherapy drugs and provide references for precise treatment. In this study, the Wilcoxon test was used for intergroup comparisons, and a *P* value < 0.05 was statistically significant.

## Results

### Identification of 610 key modules genes linked to LUAD

**Fig. 1:**
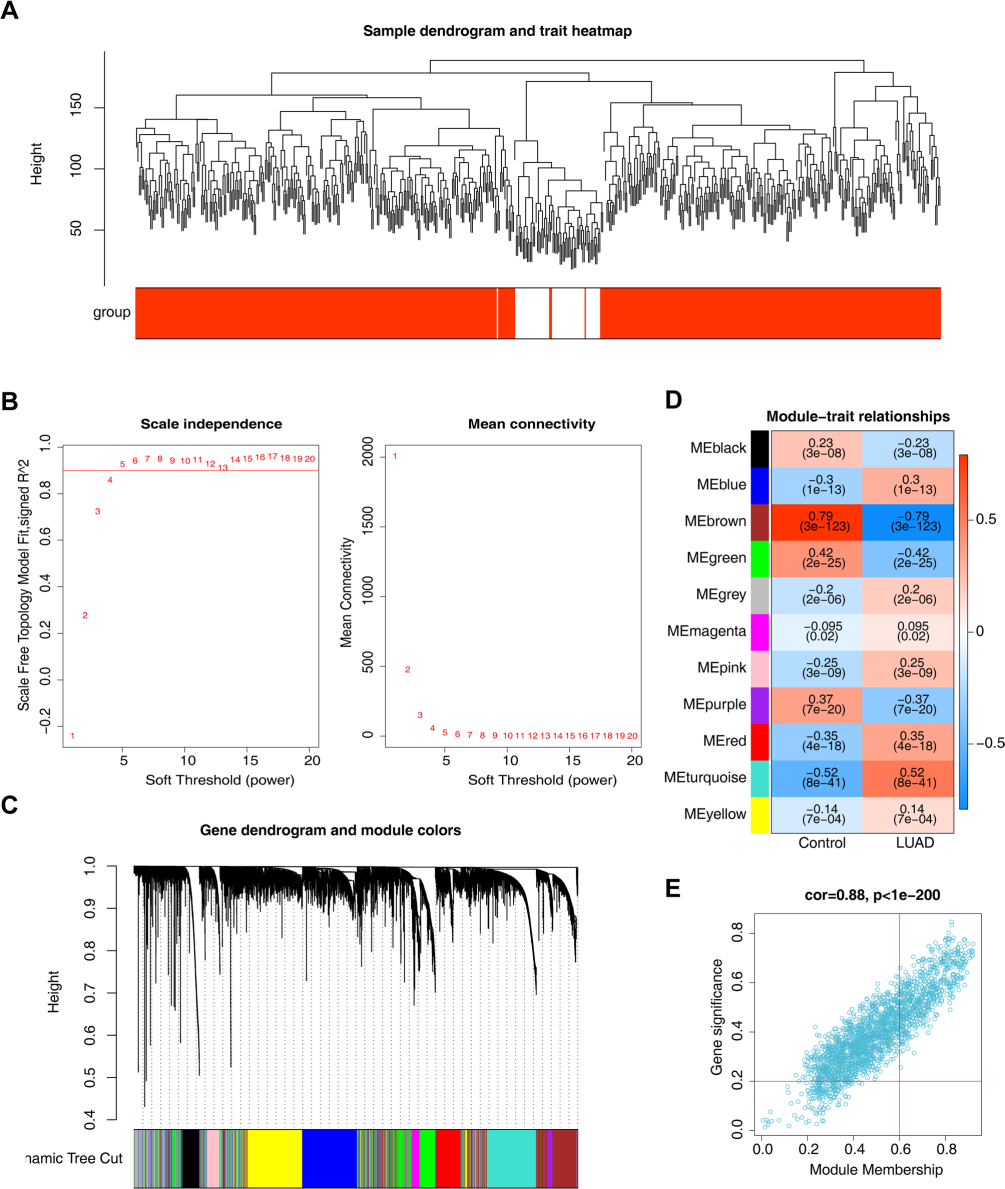
Identification of genes associated with lung adenocarcinoma (LUAD). (**A**) Clustering dendrogram of genes. (**B**) Analysis of the scale-free fit index (left) and the mean connectivity (right) for various soft-thresholding powers. (**C**) Dendrogram of all genes clustered based on a dissimilarity measure (1-TOM). (**D**) Heatmap of the correlation between the module eigengenes and clinical traits of LUAD, the color represents the Pearson correlation coefficient between the module and the trait (red: positive correlation, blue: negative correlation), and the value and the *p* value in parentheses respectively indicate the strength and significance of the correlation. Among them, the MEbrown module is strongly positively correlated with LUAD. (**E**) Scatter plot of module eigengenes in the brown module

WGCNA was employed to identify LUAD-associated genes. The clustering analysis of TCGA-LUAD samples revealed no outliers (Fig. 1A). A soft threshold of 6 was selected, where R^2^ approximated 0.9 and mean connectivity approached zero, leading to the construction of the co-expression network (Fig. 1B). Eleven modules were generated using the dynamic tree cutting method (Fig. 1C), with the Brown module exhibiting the strongest correlation with disease classification features (|r| = 0.79, *P* < 0.001) (Fig. 1D). Genes within the Brown module were screened based on|MM| > 0.6 and|GS| > 0.2, resulting in 610 genes classified as key module genes (Fig. 1E, Table S1).

### An essential connection was observed between MSCPD-RG and the occurrence of LUAD

**Fig. 2:**
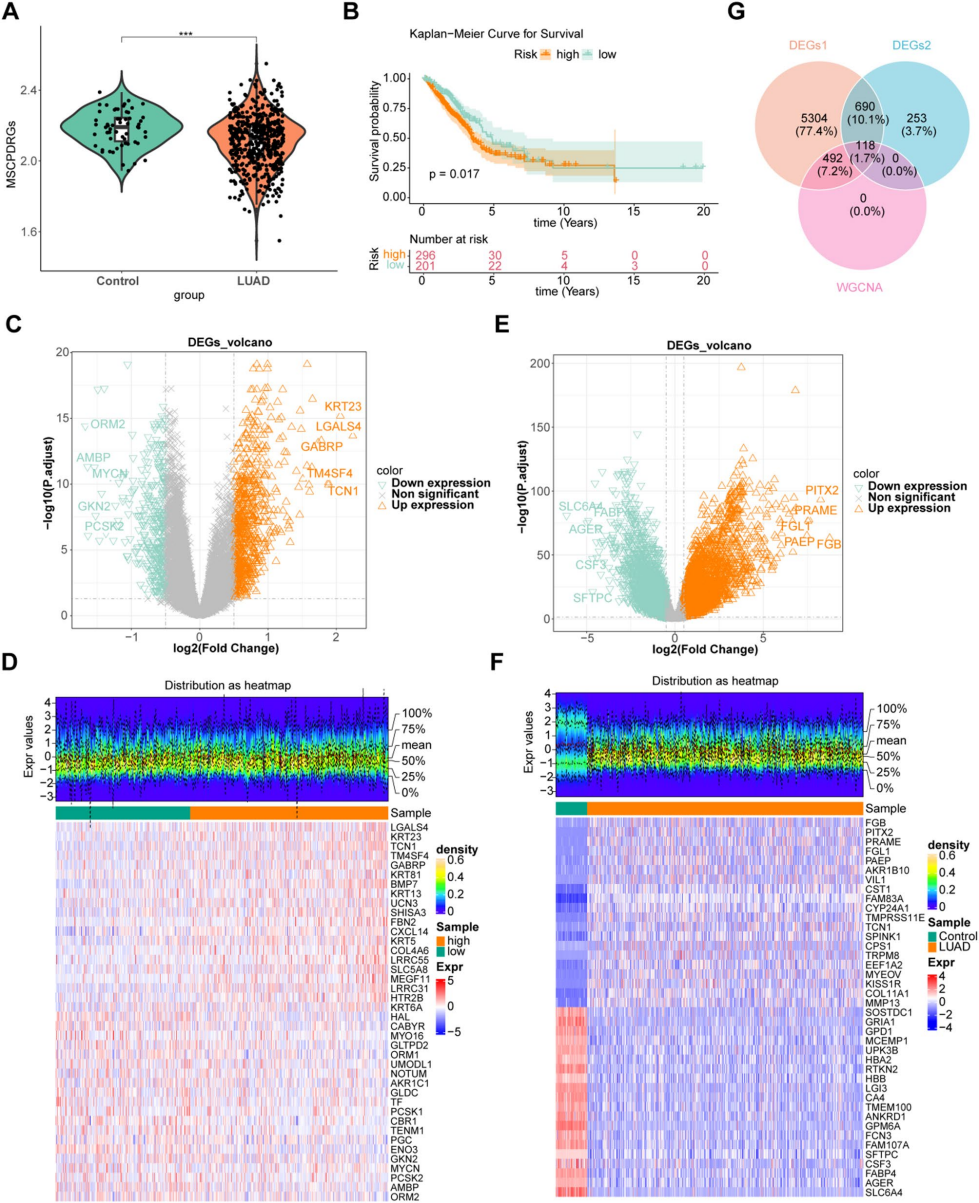
Identification of MSCP-DRGS-related genes. (**A**) MSCP-DRGS score violin chart. green represents the control group, orange represents the disease group, and *** represents *p* < 0.001. (**B**) KM curve graphs of the high and low MSCPDRGs score groups. (**C**) Volcano plots of differentially expressed genes in the high and low score groups: The orange equilateral triangle represents the up-regulated differentially expressed genes, the green inverted triangle represents the down-regulated differentially expressed genes, and the gray X-shaped genes are those without significant statistical significance. (**D**) Heat map of the expression levels of the Top20 differentially expressed genes in the low-score group. The top graph represents the density distribution of the expression levels of the differentially expressed genes. The horizontal axis of the lower graph represents the samples, and the vertical axis represents the genes. The green in the top represents the samples in the high-score group, and the orange-red represents the samples in the high-score group. In the figure, the red ones represent highly expressed genes and the blue ones represent low-expressed genes. (**E**) Volcanic map of DEGs2 between the LUAD group and the control group. (**F**) Heat map of DEGs2 between the LUAD group and the control group. (**G**) Venn diagram of the intersection of DEGs1 and DEGs2 with key module genes

To calculate MSCPD-RG scores for LUAD, computations were first conducted on all TCGA-LUAD samples. Significant differences in MSCPD-RG scores between LUAD and control groups highlighted the association between MSCPD-RGs and LUAD prevalence (Fig. 2A). The LUAD cases were subsequently divided into high and low scoring groups based on a minprop value of 0.4, revealing a significant difference in survival outcomes between the two groups (Fig. 2B).

Differential expression analysis was performed to identify DEGs1 between the two risk groups in TCGA-LUAD. A total of 1,061 DEGs1 were identified, including 701 upregulated and 360 downregulated genes (Fig. 2C and D, Table S2). Similarly, 6,604 DEGs2 were identified between the LUAD and control groups, with 3,947 upregulated and 2,657 downregulated genes (Fig. 2E and F, Table S3). Following this, 118 candidate genes were selected by overlapping DEGs1 and DEGs2 with key module genes (Fig. 2G).

### The highest binding scores were observed between FGF2-FGFR2, ROBO2-SLIT3, and ROBO2-SLIT2

**Fig. 3:**
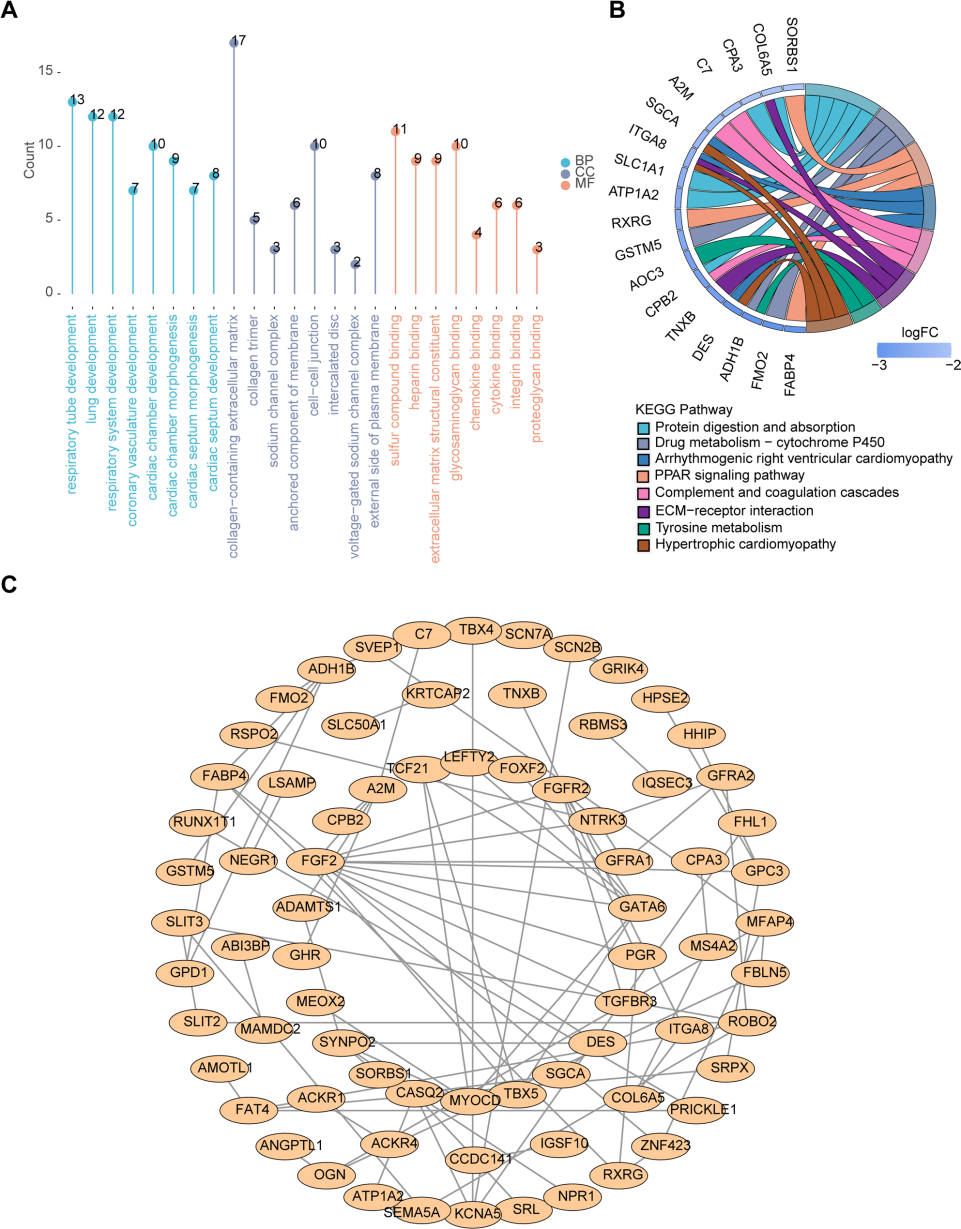
Functional enrichment analysis of genes related to lung adenocarcinoma (LUAD). (**A**) Gene Ontology (GO) enriched entries for candidate genes (top 8 of each section is shown according to *P*-value). (**B**) Kyoto Encyclopedia of Genes and Genomes (KEGG) enriched pathways for candidate genes (top 8 of pathways are shown according to *P*-value). (**C**) Interaction network of encoded proteins between candidate genes

To explore the biological pathways associated with these 118 candidate genes in LUAD, functional enrichment analysis was conducted. GO analysis indicated key associations with respiratory tube development, collagen-containing extracellular matrix, and sulfur compound binding (Fig. 3A). KEGG analysis revealed significant pathways, including protein digestion and absorption, PPAR signaling, and chemical carcinogenesis-receptor activation (Fig. 3B). Additionally, the PPI network highlighted interactions among 75 of the 118 candidate genes, with the strongest binding scores observed between FGF2-FGFR2, ROBO2-SLIT3, and ROBO2-SLIT2 (Fig. 3C).

### *MS4A2*, *IGSF10*, *NTRK3*, *MFAP3L* were identified as biomarkers of LUAD

**Fig. 4:**
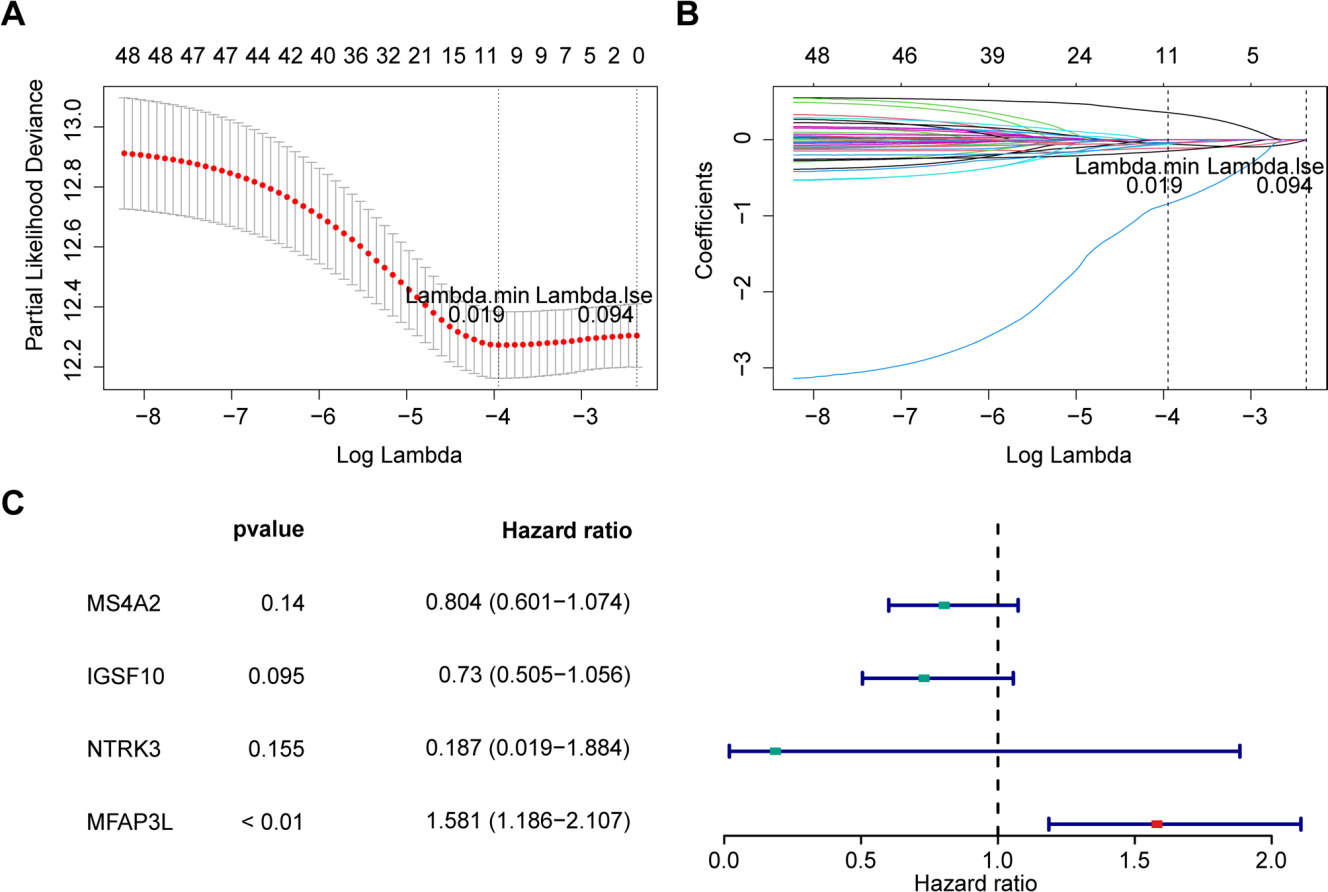
Accurate selection of biomarkers for lung adenocarcinoma (LUAD). (**A-B**) LASSO regression analysis on genes meeting the PH hypothesis test. (**C**) Multivariate cox regression analysis identifying biomarkers of LUAD survival

To identify precise biomarkers, univariate Cox regression analysis was performed to select 50 genes significantly associated with survival in TCGA-LUAD (Figure S1). Of these, 48 genes passed the PH assumption test (Table S4). Using these genes, LASSO regression was applied, with the error minimized at lambda.min = 0.019, resulting in 9 genes (*ADAMTS8*, *MS4A2*, *C1QTNF7*, *HPSE2*, *IGSF10*, *NTRK3*, COL6A5, *MFAP3L*, *FGFR2*) (Fig. 4A and B). A subsequent multivariate Cox analysis identified *MS4A2*, *IGSF10*, *NTRK3*, and *MFAP3L* as the key biomarkers (Fig. 4C).

### The predictive effectiveness of biomarker-based risk modeling was robust

**Fig. 5:**
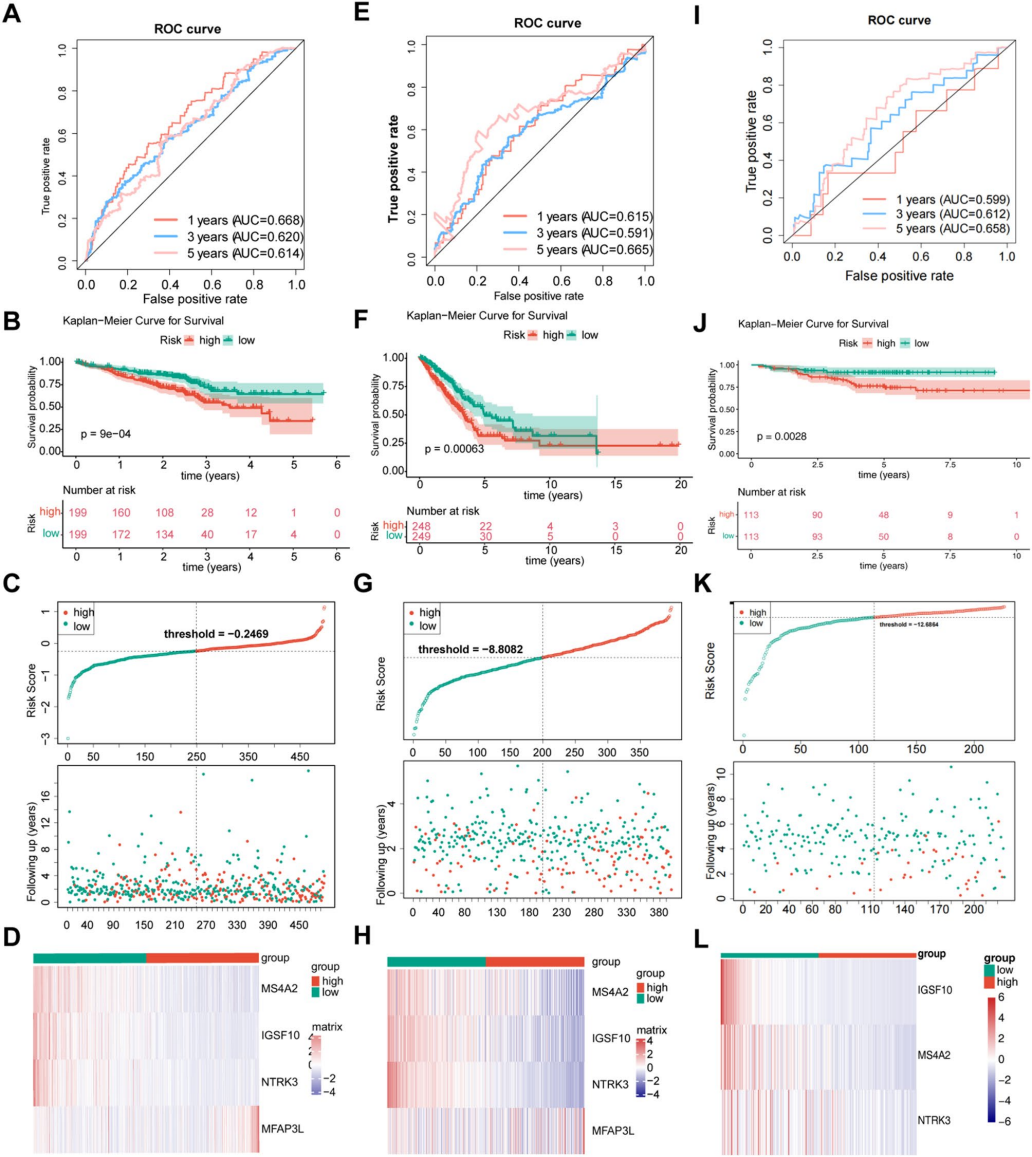
Construction of prognostic risk model (**A**) ROC curve (training set). (**B**) The KM curve of the risk model (training set), the horizontal coordinates of both the upper and lower halves of the image represent time, the vertical coordinate of the upper half represents the survival rate, the vertical coordinate of the lower half represents different groups, and the numbers represent the number of survival samples. (**C**) Risk curve (training set). (**D**) Heat maps of biomarker expression levels in the high-risk and low-risk groups (training set). (**E**) ROC curve (validation set GSE72094). (**F**) Risk model KM curve (validation set GSE72094). (**G**) Risk curve (Validation set GSE72094). (**H**) Heat maps of prognostic gene expression levels in the high-risk and low-risk groups (validation set GSE72094). (**I**) ROC curve (validation set GSE31210). (**J**) KM curve of risk model (validation set GSE31210). (**K**) Risk curve (validation set GSE31210). (**1**) Heat map of prognostic gene expression levels in high-risk and low-risk groups (validation set GSE31210 )

To further assess the impact of biomarkers, risk scores were calculated, and risk models were developed based on their expression and survival data. The AUC values of the ROC curves for patients with LUAD at 1, 3, and 5 years all exceeded 0.6, demonstrating the models’ accuracy in predicting survival outcomes for patients with LUAD (Fig. 5A). LUAD cases were then divided into high-risk (*n* = 248) and low-risk (*n* = 249) groups based on the median risk score of -0.341050913201201. Survival analysis between the two groups revealed that patients in the high-risk group had a significantly worse survival outcome compared to those in the low-risk group (*P* = 0.0063), with risk curves supporting this result (Fig. 5B and D). The GSE72094 and GSE31210 datasets further validated the prognostic efficacy of the risk model (Fig. 5E and L).

### Extent and size of primary tumor and risk score were identified as independent prognostic factors

**Fig. 6:**
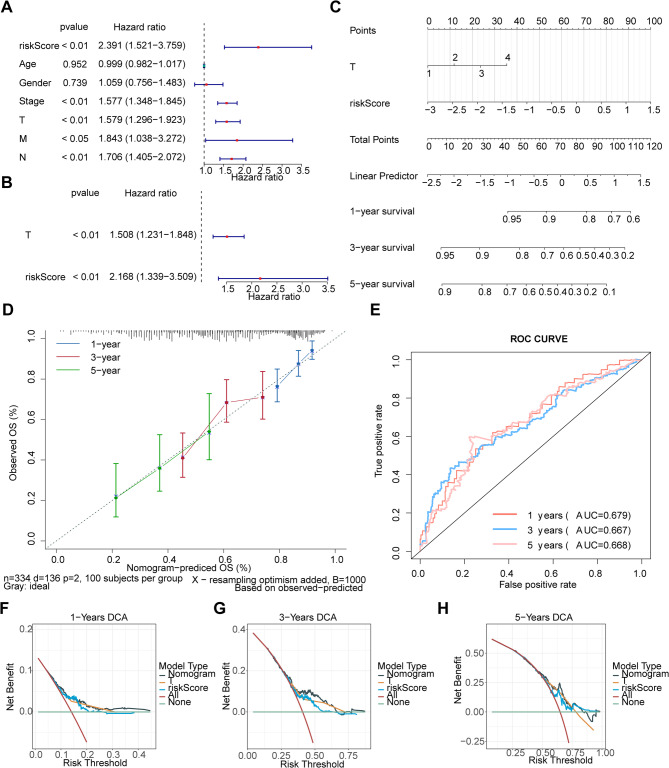
Independent prognostic analysis in lung adenocarcinoma (LUAD). (**A**) Univariate cox analysis and PH hypothesis testing of clinical factors and risk score. (**B**) Multivariate cox analysis identifying independent prognostic factors. (**C**) Nomogram model predicting survival rates. (**D**) Calibration curve of the nomogram. The abscissa represents the predicted event incidence, and the ordinate represents the actual event incidence. Both ranges are from 0 to 1, namely the event incidence (percentage). The dashed diagonal line is the reference line, indicating the case where the predicted value equals the actual value. (**E**) ROC curve of the Nomogram model. (**F**) 1-year DCA curve of the Nomogram model. The abscissa is the Risk Threshold, and the ordinate is the Net Benefit. (**G**) 2-year DCA curve of the Nomogram model. (H) 3-year DCA curve of the Nomogram model

In univariate Cox regression and PH assumption testing, clinical factors and the risk score were incorporated, revealing that T (extent and size of primary tumor), risk score, and M (distal metastasis) were consistent with the threshold criteria (Fig. 6A, Table S5). Multivariate Cox analysis of these three parameters identified T and risk score as independent prognostic factors (Fig. 6B). Based on these independent prognostic factors (T stage and risk score), a nomogram was constructed to predict the 1-, 3-, and 5-year survival rates of patients with LUAD, which indicated that higher scores were associated with lower survival rates (Fig. 6C). The nomogram demonstrated superior predictive performance, evidenced by a slope approaching one on the calibration curve, an AUC greater than 0.6 for the ROC curve, and a larger gain in DCA (Fig. 6D and H).

### Nine distinct immune cells invading two risk groups

**Fig. 7:**
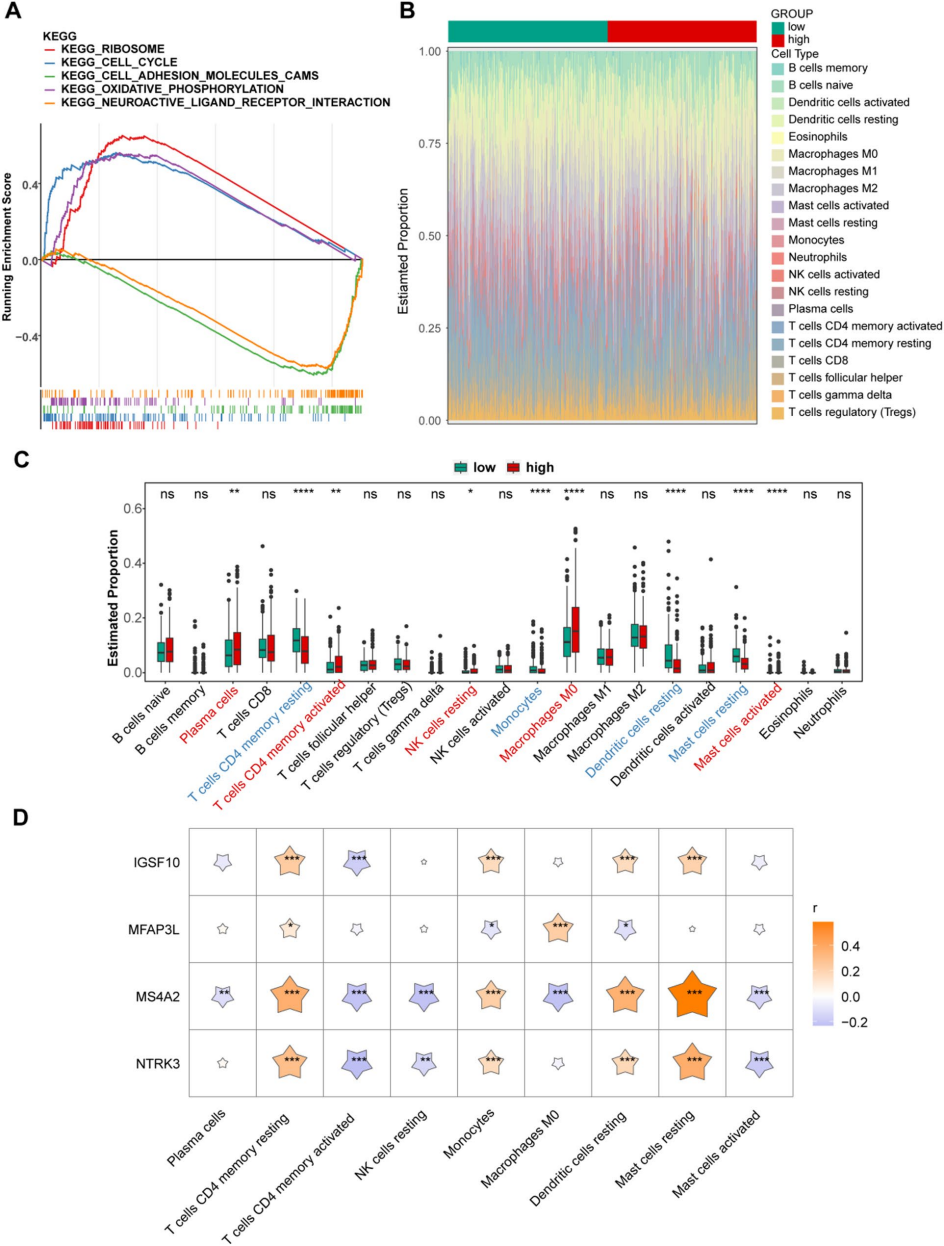
Analysis of GSEA enrichment and immune infiltration in the high-risk and low-risk groups. (**A**) Functional pathway analysis using GSEA in LUAD risk groups (top 5). (**B-C**) Immune cell infiltration analysis between LUAD risk groups. (**D**) Correlation analysis between immune cells and biomakers in LUAD

GSEA and immune infiltration analysis were performed to explore the functional pathways involved in differential gene expression between the two risk groups. Pathways such as ribosome biogenesis, the cell cycle, and oxidative phosphorylation were found to be significantly associated with LUAD progression (Fig. 7A). Immune infiltration analysis revealed substantial differences in the abundance of nine immune cell types, including plasma cells and CD4 memory resting T cells, between the two risk groups (Fig. 7B and C). *MS4A2* showed the highest correlation with resting mast cells (*r* = 0.58), while *NTRK3* had the lowest correlation with activated memory CD4 T cells (*r* = -0.23) (Fig. 7D).

### Molecular regulation of CeRNA network junction biomarkers

**Fig. 8:**
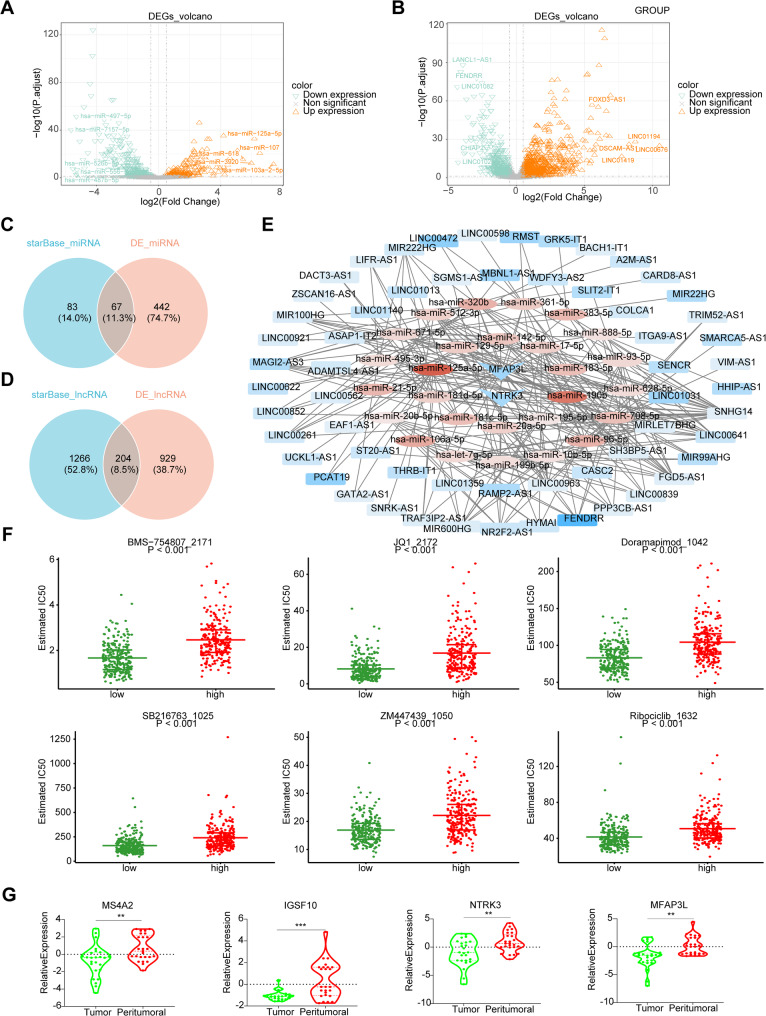
Analysis of molecular regulation and drug sensitivity in LUAD risk stratification. (**A-B**) Differential expression analysis of miRNAs and lncRNAs. (**C**) Selection of miRNAs targeted by biomarkers. (**D**) Selection of lncRNAs targeted by biomarker-associated miRNAs. (**E**) The ceRNA network construction. (**F**) Drug sensitivity analysis in LUAD risk stratification. (**G**) The qRT-PCR analysis of biomarker expression in 9 lung cancer and 36 normal tissue

ceRNA network was constructed to elucidate the molecular regulatory mechanisms underlying the biomarkers. In TCGA-LUAD, differential expression analysis identified 509 DE-miRNAs (211 upregulated and 298 downregulated) and 1,133 DE-lncRNAs (711 upregulated and 422 downregulated) (Fig. 8A and B). Subsequently, 150 miRNAs from the starBase database, predicted to target the four biomarkers, were intersected with the DE-miRNAs, resulting in 67 miRNAs (Fig. 8C). Of these, 27 miRNAs exhibiting the opposite expression trend of the biomarkers were selected as target miRNAs. Using the same approach, 27 predicted lncRNAs for these target miRNAs were identified, resulting in 204 intersecting lncRNAs (Fig. 8D), with 59 selected as target lncRNAs. A ceRNA network was then constructed, consisting of biomarkers, their targeted miRNAs, and the corresponding lncRNAs (Fig. 8E).

The comprehensive drug sensitivity analysis revealed significant differences in the IC_50_ values of 117 drugs between the two risk groups. Six drugs demonstrated higher efficacy in the low-risk group: BMS-754807_2171, JQ1_2172, Doramapimod_1042, SB216763_1025, ZM447439_1050, and Ribociclib_1632 (Fig. 8F).

### The expression of biomarkers in lung cancer and normal lung tissue

Furthermore, the expression of the four biomarkers was assessed in clinical lung cancer and normal lung tissues using qRT-PCR. The results showed that the expression of all four biomarkers was significantly downregulated in lung cancer tissues compared to normal tissues (Fig. 8G).

## Discussion

LUAD, the most prevalent subtype of lung cancer, is distinguished by its high metastatic potential and rapid proliferation, which often lead to a poor overall prognosis. Despite advances in diagnostic and therapeutic strategies, the clinical management of LUAD remains challenging due to the lack of reliable prognostic indicators capable of accurately predicting patient outcomes. The proliferation and differentiation of MSCs play a critical role in tumor development, particularly in the malignant progression of LUAD [37]. This study utilized bioinformatics analysis to identify genes associated with MSC proliferation and differentiation in LUAD, examining their roles in gene expression, prognosis, immune infiltration, and drug sensitivity. Ultimately, a prognostic risk model incorporating MS4A2, IGSF10, NTRK3, and MFAP3L was developed to predict the outcomes of patients with LUAD. This methodological approach aligns with contemporary lung cancer research paradigms. For instance, Zhang et al. successfully delineated genetic overlap between lung cancer and gastroesophageal reflux disease through integrated systems biology and machine learning [38], while Wang et al. established a four-gene model from GEO data that validated differentially expressed genes’ predictive capacity for tumor microenvironment immune infiltration [10]. Notably, the MSCPD risk model developed in this study demonstrates dual methodological and clinical advantages. Focusing specifically on MSCPD-related gene prognostic modeling, our work not only confirms the clinical significance of four pivotal genes (including MS4A2) across multiple TCGA and GEO cohorts, but also uncovers a unique tumor progression mechanism mediated through mesenchymal-epithelial transition regulation. These findings complement the independent prognostic role of SMAD7 reported by Chen et al. [39], collectively advancing the theoretical framework for LUAD molecular classification.

MS4A2, a member of the MS4A protein family, is primarily expressed on mast cells and basophils [40]. Within the tumor microenvironment, these cells release various immune mediators that influence both immune and non-immune components, modulating the immune response [41]. Numerous studies have underscored the prognostic relevance of MS4A2 across various cancers, including gastric [42, 43], colorectal [44], and LUAD [45]. MS4A2 has emerged as an independent prognostic factor in LUAD, even in early stages [46], potentially linked to mast cell activity within the tumor microenvironment. Studies demonstrate that mast cell-derived cytokines (including TGF-β and IL-6) critically promote MSCs differentiation into cancer-associated fibroblasts [16]. This mechanism elucidates how MS4A2 regulates tumor progression through mast cell-mediated MSC interactions. Furthermore, MS4A2 represents a promising autonomous prognostic factor and therapeutic target for enhancing anti-tumor immunity, especially in early-stage disease. In our analysis utilizing GSEA and immune infiltration techniques, a significant correlation was observed between MS4A2 and resting mast cells, supporting the hypothesis that mast cells play a pivotal role in LUAD progression. One study reported that, as LUAD progresses, mast cells, monocytes, and lymphatic endothelial cells gradually decrease, correlating with increased invasiveness of LUAD [47]. Further exploration of MS4A2’s role in LUAD holds significant potential for advancing future research.

IGSF10, a member of the immunoglobulin superfamily, is expressed at low levels in various cancers, including lung, breast, colon, and head and neck cancers [48]. In breast cancer, IGSF10 plays a role in DNA repair, cell cycle regulation, and glycolysis, and is associated with the PI3K/Akt/mTOR and mTORC1 signaling pathways [49]. Knocking out IGSF10 significantly enhances lung cancer cell proliferation, promotes cell-matrix adhesion, and activates the integrin-β1/FAK pathway [50]. This activation is marked by increased expression of integrin-β1, p-FAK, and p-AKT, illustrating IGSF10’s role in inhibiting lung cancer metastasis and invasion. Additionally, IGSF10 suppresses LUAD cell metastasis through the Spi-B/Integrin-β1 signaling pathway [51]. IGSF10 inhibits LUAD cell migration via the Spi-B/Integrin-β1 signaling pathway [51], disrupts normal mesenchymal stromal cell (MSC) differentiation, and induces tumor microenvironment imbalance, and regulation of IGSF10 expression by miR-106b-5p in LUAD tissues emphasizes its role in inhibiting metastasis and invasion, suggesting potential therapeutic interventions [52]. As a prognostic marker across various cancers, further research on IGSF10 is essential and may uncover additional therapeutic avenues.

NTRK3, encoding a neurotrophic factor receptor, is vital for neuronal development, growth, and survival [53]. The neurotrophin receptor encoded by NTRK3 participates not only in neuronal development but also critically regulates osteogenic differentiation of MSCs [54], indicating its potential role in tumor matrix remodeling through modulation of MSC differentiation fates. NTRK3 gene fusion, which results from intrachromosomal or interchromosomal rearrangements, drives oncogenesis [55]. These fusion events promote tumor proliferation and spread, and targeted therapies like TRK inhibitors have proven effective in addressing these fusions [56]. Common mutations in NTRK3 in LUAD can predict patient prognosis during immune checkpoint inhibitor (ICI) therapy, offering significant clinical implications for immunotherapy [57]. In the present study, NTRK3 exhibited a negative correlation with activated memory CD4 T cells. Although further studies are needed, existing research has highlighted the interaction between NTRK3 and various immune lymphocytes [58]. Given its established role as a therapeutic target, future research could explore NTRK3’s dynamic alterations throughout LUAD onset and progression to address the disease at different stages.

MFAP3L, a microfibrillar protein involved in tumorigenesis, functions as a kinase when activated, phosphorylating ERK2 at Tyr187. MFAP3L regulates MSC proliferation through the ERK signaling pathway [59], consistent with prior studies demonstrating that ERK pathway activation enhances MSC migration ability. Overexpression of MFAP3L initiates the ERK signaling cascade, promoting the expression of ERK regulatory genes and enhancing metastasis and invasion in colon cancer cells [59]. miR-671-5p negatively regulates MFAP3L by targeting its 3’-UTR, thereby inhibiting proliferation, migration, and invasion in NSCLC cells [60]. Targeting the miR-671-5p/MFAP3L signaling pathway presents a promising strategy to curb NSCLC progression. Given the correlation between MFAP3L and LUAD, further investigation is warranted, potentially opening up new therapeutic approaches. This study confirmed significant downregulation of four MSCPD-related biomarkers (MS4A2, IGSF10, NTRK3, and MFAP3L) in LUAD tissues via qRT-PCR (*p* < 0.01). This finding showed high consistency with TCGA transcriptome data (log2FC = -1.2∼-2.8), validating the reliability of bioinformatics analysis.

Recent advances demonstrate significant efficacy of immunotherapy in lung cancer treatment [61]. The four MSCPD-related biomarkers (MS4A2, IGSF10, NTRK3, and MFAP3L) identified in this study provide multidimensional insights for LUAD immunotherapy. From the metabolic regulation perspective, MS4A2 modulates the tumor immune microenvironment through mast cell activity, with its high correlation to resting mast cells (*r* = 0.58) suggesting predictive potential for immunotherapy response - analogous to mast cell markers in melanoma [62] and aligning with discoveries on metabolic reprogramming in CAR-T efficacy [63]. IGSF10 regulates T cell function via the Spi-B/Integrin-β1 pathway, where its low expression correlates with poor prognosis, mirroring PD-1/PD-L1 dynamics in gastric cancer immunotherapy [64]. NTRK3 gene fusion serves not only as a targeted therapy target but also shows negative correlation with memory CD4 + T cells (*r*=-0.23), suggesting immunotherapy impact through altered T cell metabolism [63]. Notably, tumor microenvironment MSCs may shape immunosuppression via exosome-mediated communication [65], requiring clinical validation. Clinically, these markers show liquid biopsy potential: ctDNA-based detection monitors immune microenvironment dynamics [66], IGSF10 promoter methylation may enable non-invasive early diagnosis [67], and peripheral blood NTRK3 fusion detection correlates with tumor burden [56], paralleling molecular barcode monitoring in pancreatic/biliary malignancies [68]. Future studies should integrate single-cell metabolomics and exosome analysis to dynamically monitor biomarker heterogeneity, optimizing next-generation CAR-T and immune checkpoint therapies.

Immune profiling revealed significant associations between MSCPD-related genes and specific immune cell subsets: high-risk LUAD patients exhibited reduced plasma cell (*p* = 0.003) and CD4 + memory resting T cell infiltration (*p* = 0.008), alongside increased M2 macrophages (*p* = 0.012). These findings carry important biological implications: plasma cell reduction may impair antibody-dependent tumor immunity [69], while CD4 + memory T cell depletion correlates with IGSF10-mediated integrin β1 signaling abnormalities. ceRNA network analysis identified 27 key miRNAs and 59 lncRNAs, with the miR-671-5p/MFAP3L [10] and miR-106b-5p/IGSF10 [70] regulatory networks being particularly prominent - both known to regulate MSC differentiation and immune microenvironment remodeling. Notably, lncRNA MALAT1 may indirectly modulate NTRK3 expression by sponging miR-200 family members [39], thereby altering MSC neural differentiation potential. These results provide new molecular perspectives for understanding LUAD progression.

Functional enrichment analysis revealed significant enrichment of MSCPD-related genes in ribosome, cell cycle, and oxidative phosphorylation pathways between high- and low-risk LUAD groups. Ribosome pathway activation enhances protein synthesis efficiency in MSCs and tumor cells, promoting MSC differentiation into CAFs and tumor microenvironment remodeling through its signaling axis [71, 72]; cell cycle enrichment (e.g., CDK1, CCNB1) releases cycle inhibition to enable sustained proliferation of MSCs and LUAD cells [73]; while oxidative phosphorylation enrichment indicates MSCPD provides energy support for LUAD via mitochondrial metabolic reprogramming. GSEA analysis further demonstrated that biomarkers NTRK3 and MFAP3L are respectively regulated by these pathways, ultimately coordinating LUAD progression.

The MSCPD-related gene prognostic model for lung adenocarcinoma proposed in this study demonstrates unique advantages and complementary value compared to existing models. Relative to T cell marker models [74], our approach not only addresses immune microenvironment regulation (e.g., MS4A2-mediated mast cell activity) but also shows superior performance in early metastasis risk prediction. Compared with copper-death models reflecting metabolic stress [75], our model captures broader pro-metastatic mechanisms through IGSF10 and NTRK3. Clinically, our markers enable stable detection from routine FFPE tissues or trace plasma samples. When benchmarked against circulating tumor cell models (1-/3-/5-year AUCs: 0.68/0.64/0.58; [76]), our model demonstrates more stable performance (AUCs: 0.67/0.62/0.61), greater operational feasibility, and enables dynamic monitoring through continuous scoring - providing real-time clinical decision support. This rigorous methodology has resulted in a highly accurate model, demonstrating outstanding performance in forecasting survival outcomes at 1, 3, and 5 years. The study underscores the critical role of primary tumor extent and size, in addition to the risk score derived from biomarker analysis, as pivotal factors in determining prognosis. Furthermore, the molecular signatures identified through this biomarker analysis significantly enhance the understanding of prognosis, enabling a more comprehensive evaluation of disease progression. These findings not only validate the robustness of our model but also highlight its superiority in providing actionable insights for personalized patient care.

This study examined the expression levels and regulatory mechanisms of MSCPD-related genes in LUAD, identifying four key biomarkers—MS4A2, IGSF10, NTRK3, and MFAP3L—and conducting detailed functional enrichment and immune-related analyses on these candidates. These analyses present a novel avenue for understanding the regulatory mechanisms of MSCPD in LUAD. However, this study has limitations. Although bioinformatics analysis and qRT-PCR confirmed expression differences in MS4A2, IGSF10, NTRK3, and MFAP3L, functional experiments are needed to elucidate how these genes specifically regulate mesenchymal stem cell proliferation/differentiation and their exact mechanisms in LUAD progression. Crucially, whether these genes influence tumor development through established pathways—FcεRI signaling (MS4A2), Integrin-β1/FAK (IGSF10), or ERK transduction (MFAP3L)—requires further validation, as does their relationship with MSC activity. Additionally, despite rigorous batch-effect correction, residual technical variations from heterogeneous sample sources, sequencing platforms, and population characteristics across datasets may affect analytical practicality. Finally, the limited qRT-PCR validation sample size impacts result reliability. Future work will: (1) establish gene-edited animal models (e.g., conditional knockouts) for in vivo functional verification; (2) employ co-culture systems simulating tumor microenvironments to analyze MSC differentiation regulation; and (3) conduct prospective clinical studies evaluating liquid biopsy utility while expanding sample cohorts. These efforts will advance translation of findings into clinically valuable diagnostic/therapeutic strategies.

## Conclusion

In this study, four biomarkers associated with MSCPD were identified as reliable prognostic predictors for patients with LUAD. A comprehensive bioinformatics analysis confirmed the strong correlation between MSCPD and LUAD, offering new perspectives on diagnosing and treating individuals with this condition. These findings provide a robust foundation for advancing clinical strategies and offer valuable directions for future research.

## Electronic supplementary material

Below is the link to the electronic supplementary material.

Supplementary Material 1

Supplementary Material 2

Supplementary Material 3

Supplementary Material 4

Supplementary Material 5

Supplementary Material 6

## Data Availability

The datasets used and/or analyzed during the current study are available from the corresponding author on reasonable request.

## Abbreviations

MSCs: Mesenchymal stem cells
LUAD: Lung adenocarcinoma
DEGs: Differentially expressed genes
NSCLC: Non-small cell lung cancer
TCGA: The cancer genome atlas
GEO: Gene expression omnibus
MSigDB: Molecular signatures database
WGCNA: Weighted gene co-expression network analysis
GS: Gene significance
ssGSEA: Sample gene set enrichment analysis
KM: Kaplan-meier
GO: Gene ontology
KEGG: Kyoto encyclopedia of genes and genomes
ROC: Receiver operating characteristic
AUC: Area under curve
DCA: Decision curve analysis
NES: Normalized enrichment score
GDSC: Drug sensitivity in cancer
LASSO: Least absolute shrinkage and selection operator
qRT-PCR: Quantitative real-time polymerase chain reaction
