# Supplementary figures and images for: Identification of biomarkers associated with proliferation and differentiation of mesenchymal stem cells in pulmonary adenocarcinoma and establishment of prognostic models
Source: Hereditas. 2025 Jul 1;162:118. doi: 10.1186/s41065-025-00492-7 (PMC12220362; doi:10.1186/s41065-025-00492-7)

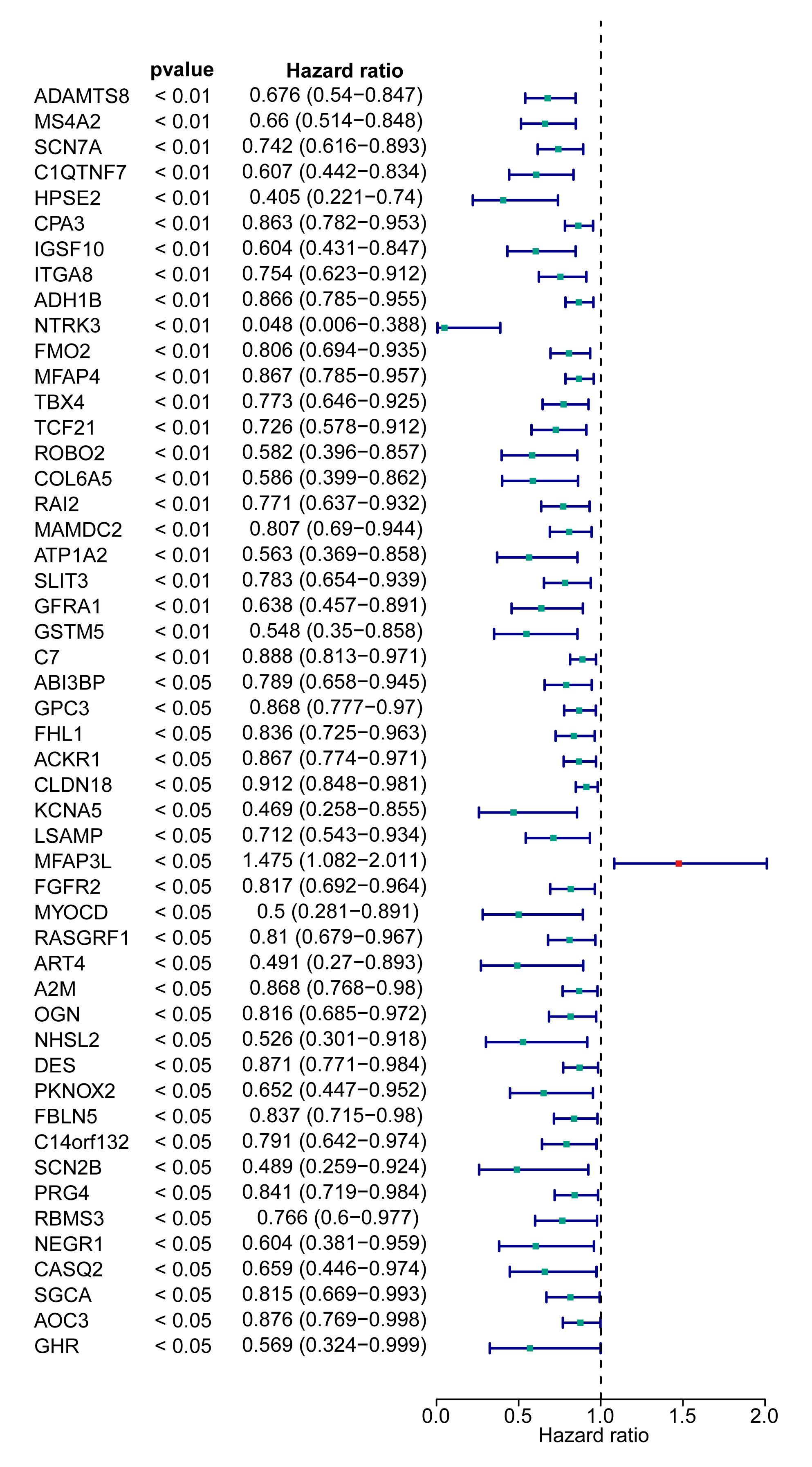

Supplement: Supplementary file 6 — Supplementary Material 6 [file 41065_2025_492_MOESM6_ESM.png]
